# Effectiveness of an Interactive Digital Intervention Program on Knowledge, Health Literacy, and Learner Engagement in Senior High School Students: Intragroup and Intergroup Comparison of 2 Teaching Models

**DOI:** 10.2196/76109

**Published:** 2025-09-03

**Authors:** Yih-Ming Weng, Yi-Xuan Li, Ching-Hao Chang, Su-Fei Huang, Jung-Yu Liao, Kuei-Yu Huang, Hsiao-Pei Hsu, Chiu-Mieh Huang, Jong Long Guo

**Affiliations:** 1 Department of Health Promotion and Health Education College of Education National Taiwan Normal University Taipei Taiwan; 2 Tri-Service General Hospital, Tingzhou Branch Taipei Taiwan; 3 Division of Medicinal Products Taiwan Food and Drug Administration Ministry of Health and Welfare Taipei Taiwan; 4 Department of Intelligent Technology and Long-Term Care MacKay Junior College of Medicine, Nursing, and Management Taipei Taiwan; 5 Institute of Traditional Medicine National Yang Ming Chiao Tung University Taipei Taiwan; 6 Department of Chinese Medicine Shin Kong Wu Ho Su Memorial Hospital Taipei Taiwan; 7 Department of Nursing, College of Nursing National Yang Ming Chiao Tung University Taipei Taiwan; 8 Institute of Clinical Nursing, College of Nursing National Yang Ming Chiao Tung University Taipei Taiwan

**Keywords:** interactive digital intervention, online program, health literacy, learner engagement, illegal drugs, adolescents, substance use prevention

## Abstract

**Background:**

Adolescent substance use remains a critical and persistent public health concern worldwide. The initiation of drug use during adolescence is often associated with long-term negative health, social, and academic outcomes. As structured environments where young people spend a large portion of their time, schools are in a unique position to implement early prevention strategies to effectively address this issue. In recent years, digital technologies have emerged as promising tools for delivering health education in engaging and scalable ways.

**Objective:**

This study aimed to develop and evaluate an interactive digital intervention (IDI) program designed to improve high school students’ knowledge related to illegal drugs, enhance their health literacy, and promote greater learner engagement. Specifically, the study sought to compare the outcomes of a digital teaching model with those of traditional textbook-based instruction.

**Methods:**

A quasi-experimental, pre-post design was adopted involving 768 senior high school students aged between 16 and 18 years from 9 randomly selected schools. The schools were assigned to either the IDI group (n=379, 49.3%) or the traditional didactic (TD) group (n=389, 50.7%). The IDI group received a 6-unit web-based substance use prevention program with interactive features such as videos, quizzes, and scenario-based discussions. The TD group received conventional classroom instruction using standard textbooks. After accounting for student attrition and absences, 651 students remained for final analysis (IDI: n=305, 46.9%; TD: n=346, 53.1%). Paired *t* tests and generalized estimating equations (GEEs) were used to assess within- and between-group differences, adjusting for age and gender.

**Results:**

An intragroup comparison revealed that the IDI group had significantly greater improvements in the following variables: knowledge (t_304_=–5.23, *P*<.01), health literacy (t_304_=–3.18, *P*<.01), functional literacy (t_304_=–3.50, *P*<.01), critical literacy (t_304_=–2.79, *P*=.01), communicative literacy (t_304_=–2.26, *P*=.02), and learner engagement (t_304_=–3.40, *P*<.01), including cognitive engagement (t_304_=–2.20, *P*=.03) and emotional engagement (t_304_=–3.84, *P*<.01). While most results were consistent across paired *t* test and GEE analyses, cognitive engagement did not show significant intergroup differences.

**Conclusions:**

Our findings support the potential of IDIs for improving knowledge, health literacy, and engagement among high school students. Compared to traditional instruction, digital programs may provide a more engaging and impactful method for substance use prevention in educational settings. However, the intervention did not significantly improve all targeted outcomes, such as refusal skills and perceived harmfulness, suggesting the need for refined targeting and longer implementation periods. Further studies may explore the long-term behavioral outcomes and the scalability of such interventions.

## Introduction

### Background

Adolescent substance use is a growing global public health concern. According to the World Drug Report 2024 published by the United Nations Office on Drugs and Crime, an estimated 292 million people used drugs in 2022, accounting for 5.6% of the global population aged between 15 and 64 years. Alarmingly, cannabis use specifically is more prevalent among adolescents aged between 15 and 16 than among adults, with a usage rate of 5.5% versus 4.4% respectively, according to the World Drug Report 2024. During adolescence, the brain is still developing, and early exposure to addictive substances may increase the risk of long-term cognitive and behavioral consequences, including the rapid development of dependency [1].

In Taiwan, the 2018 National Survey on Substance Use reported a 1.15% lifetime prevalence of illicit drug use among individuals aged between 12 and 64 who could identify the type of drug used. When including modified synthetic substances—many of which could not be clearly identified—the rate increased to 1.46%, with younger populations more likely to be influenced. Among illicit drug types, amphetamine was the most commonly used, followed by ketamine. Another study by Liao et al (2018) [2] indicated that the prevalence rate of illicit drug lifetime use was 2.79%, varying between 1.18% and 1.89% depending on the type of illicit drug, among high school and vocational school students. Moreover, there was no gender difference in usage, suggesting that prevention efforts should be universally applied to all students [3]. According to 2024 statistics from the Taiwan Food and Drug Administration and the Ministry of Education, senior high school and vocational students accounted for 49.4% of all reported drug abuse cases among students across various educational levels [4], highlighting students aged between 16 and 18 years as a critical population for substance use prevention.

Adolescence during high school represents a critical transitional period that may increase the risk of illicit substance use. A review of the literature indicates that previous interventions aimed at preventing illicit and nonmedical drug use have focused more on junior high school students than on high school students [5]. Substance use among adolescents is not only a personal health concern but also a broader societal issue that contributes to academic challenges, interpersonal conflicts, criminal behavior, and an increased risk of suicide [6-8]. Research indicates that peer influence, curiosity, and resistance to authority are significant drivers of adolescent drug experimentation and use [9,10]. These factors underscore the need for early, school-based interventions tailored to the developmental and social context of high school students [11].

### Theoretical Framework

Health education programs grounded in life skills theory and health literacy are widely recognized as effective strategies for preventing substance use. Life skills refer to psychosocial competencies that enable individuals to effectively manage everyday challenges, regulate emotions, and maintain both mental and physical well-being. Training in life skills can enhance adolescents’ resilience, promote positive decision-making, and equip them with refusal strategies to resist peer pressure and external temptations [12-14]. A randomized trial of a school-based prevention program that incorporated life skills training and strengthened drug refusal skills demonstrated long-term effects on illicit drug use behaviors [15]. These findings support the effectiveness of integrating life skills into illicit drug use prevention programs for adolescents.

Health literacy is another essential component in prevention efforts. It refers to the ability to access, understand, evaluate, and apply health-related information to make informed decisions and engage in health-promoting behaviors [16]. Various definitions of health literacy have been found [17-19]. This study categorized health literacy into 3 types according to Nutbeam’s [20] framework: functional literacy (basic comprehension of health information), communicative literacy (the ability to engage in dialogue and interpret information), and critical literacy (the capacity to critically assess and apply information to one's life). Substance use prevention programs that incorporate health literacy principles have been demonstrated to enhance students' ability to make informed decisions and reduce engagement in risky behaviors [21,22].

With the rapid advancement of educational technology, the learning experience has evolved beyond traditional textbook-based instruction. Digital education platforms, including e-learning modules, augmented reality, and serious games, offer innovative, scalable, and interactive tools for health education. These platforms enhance accessibility, facilitate real-time progress tracking, and boost student engagement through immersive and contextually rich content. In particular, interactive digital learning promotes knowledge retention through sensory stimulation, situational immersion, and emotional rewards, which may positively influence students’ motivation and behavioral outcomes [23-25].

Interactive digital learning may enhance learning motivation that is appropriate for students to learn individual-level factors (eg, knowledge and perceived harmfulness) and develop competencies to manage social influences (eg, coping with peer pressure and strengthening drug refusal skills) related to illicit drug use [26,27]. In this study, interactive digital learning was integrated with life skills and health literacy approaches, based on its potential to enhance learning motivation. Game-based interactive digital interventions (IDIs) were used to promote life skills and effectively deliver serious health messages.

Research has supported that integrating real-life scenarios into digital drug education programs can help students better understand the consequences of substance use, apply knowledge to their daily lives, and increase participation in classroom discussions and activities [27-29]. Additionally, interactive learning formats have been associated with higher levels of cognitive and emotional engagement, both of which are predictors of academic success and long-term behavioral change [30-33]. Another relevant concept is the gateway theory, which posits that the use of legal substances such as tobacco, alcohol, or betel nut often precedes illicit drug use. Adolescents' perception of the harmfulness of these gateway substances can influence their decisions regarding more illegal drug use [34-37].

### Study Goal

This study aimed to evaluate the effectiveness of an interactive digital substance use prevention program designed for senior high school students. To evaluate the effectiveness of the IDI, both intragroup and intergroup comparisons were conducted between the 2 instructional models. The program integrates critical constructs, including health literacy—encompassing functional, cognitive/interactional, and critical literacy skills—life skills, particularly refusal skills, perceived harmfulness, refusal skills, and learner engagement. By combining interactive digital content with theoretical frameworks, the intervention aims to offer a comprehensive, engaging, and developmentally appropriate approach for preventing adolescent substance use.

## Methods

### Study Design and Participants

**Figure 1:**
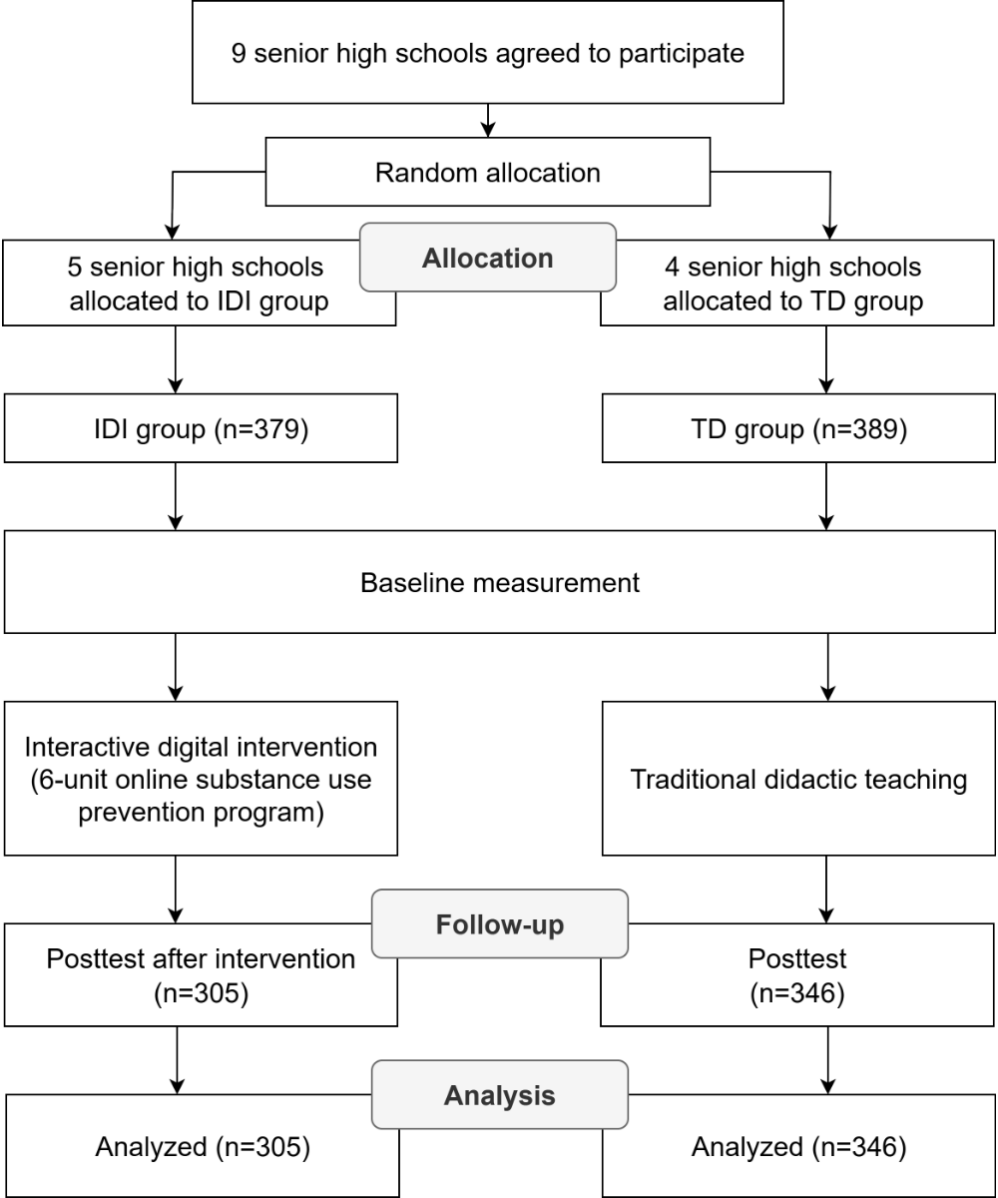
Flowchart of participants' enrollment and assessment. IDI: interactive digital intervention; TD: traditional didactic.

This study adopted a 2-arm design to evaluate the effectiveness of an IDI for substance use prevention. Schools were invited to participate based on a list published by the Ministry of Education [38] and contacted individually through formal outreach. A total of 9 senior high schools voluntarily agreed to participate. The schools were randomly allocated by an independent researcher using computer-generated random sequences, and assignments were securely stored, concealed from schools and researchers, until each school’s participation was confirmed. 49.3%. The flowchart of this study is presented in Figure 1.

A priori power analysis using G*Power software (Heinrich Heine University Düsseldorf) indicated that a sample size of 128 participants per group would be sufficient to detect a moderate effect size (Cohen *d*=0.5) with 80% power and α=.05. The final sample size of 305 (IDI) and 346 (TD) thus exceeded the required number for detecting moderate intervention effects. Figure 1 presents the flowchart of participants' enrollment and assessment.

The IDI group received a 6-unit IDI, while the TD Group received a standard textbook-based drug education curriculum without interactive digital content. All participants completed baseline and postintervention assessments.

### Intervention Program and delivery

The IDI was co-developed by professionals in health education with expertise in health education, illegal drug use prevention, nursing, and school counseling. This program includes 6 units, each incorporating interactive e-books complemented by responsive PowerPoint presentations (Microsoft Corp), learning worksheets, animated videos, and game-based modules on crucial topics, such as the risks of addictive substances, refusal skills, and critical thinking strategies. Game elements include escape rooms, scenario-based decision-making exercises, interactive quizzes, and role-playing activities.

**Figure 2:**
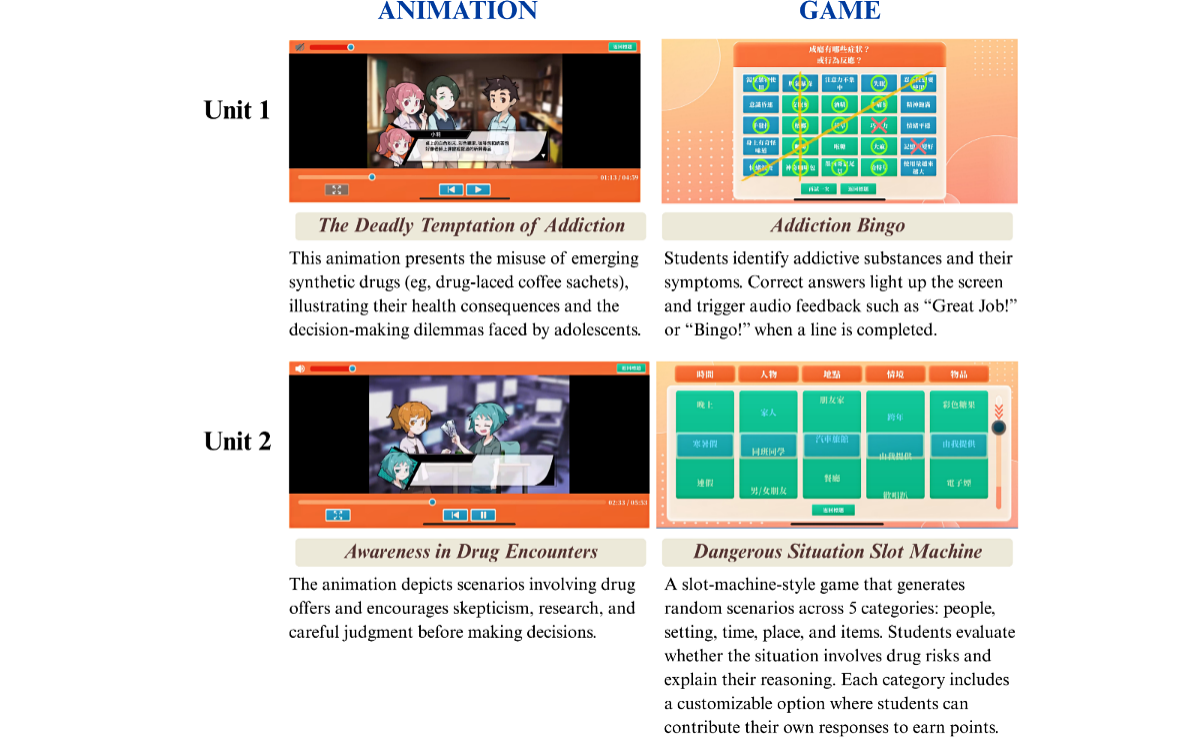
Screenshots of animations and corresponding web-based games from the interactive digital intervention (IDI) program (Units 1-2).

**Figure 3:**
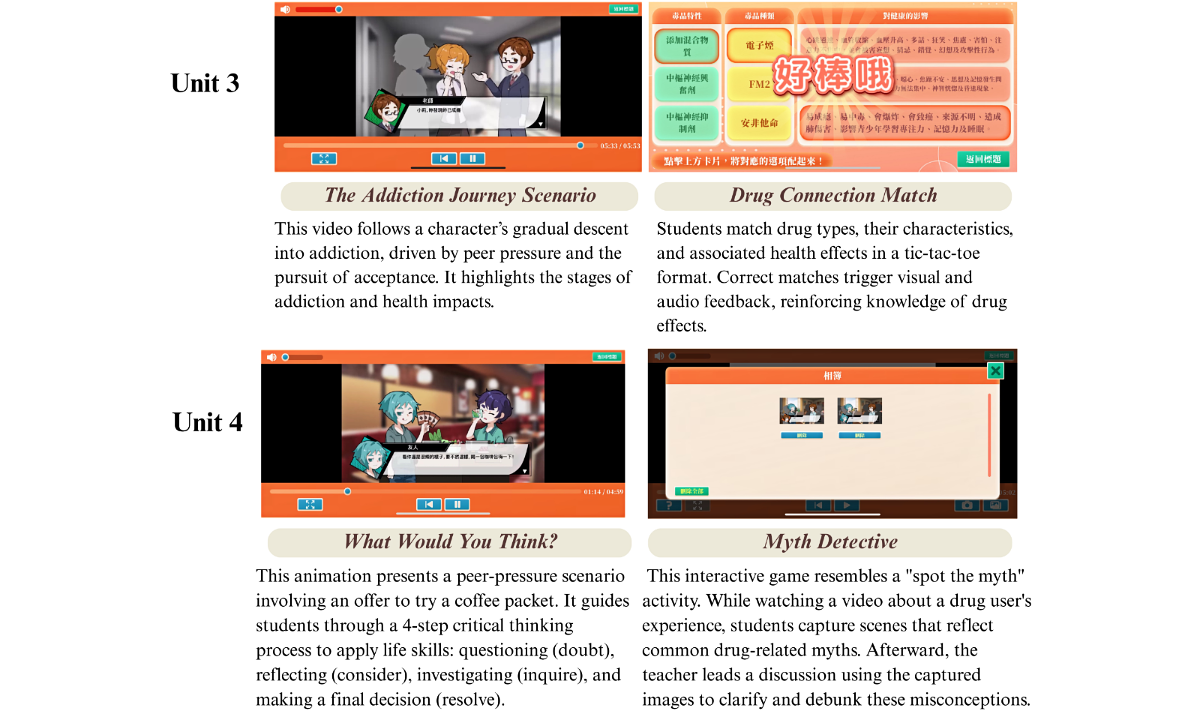
Screenshots of animations and corresponding web-based games from the interactive digital intervention (IDI) program (Units 3-4).

**Figure 4:**
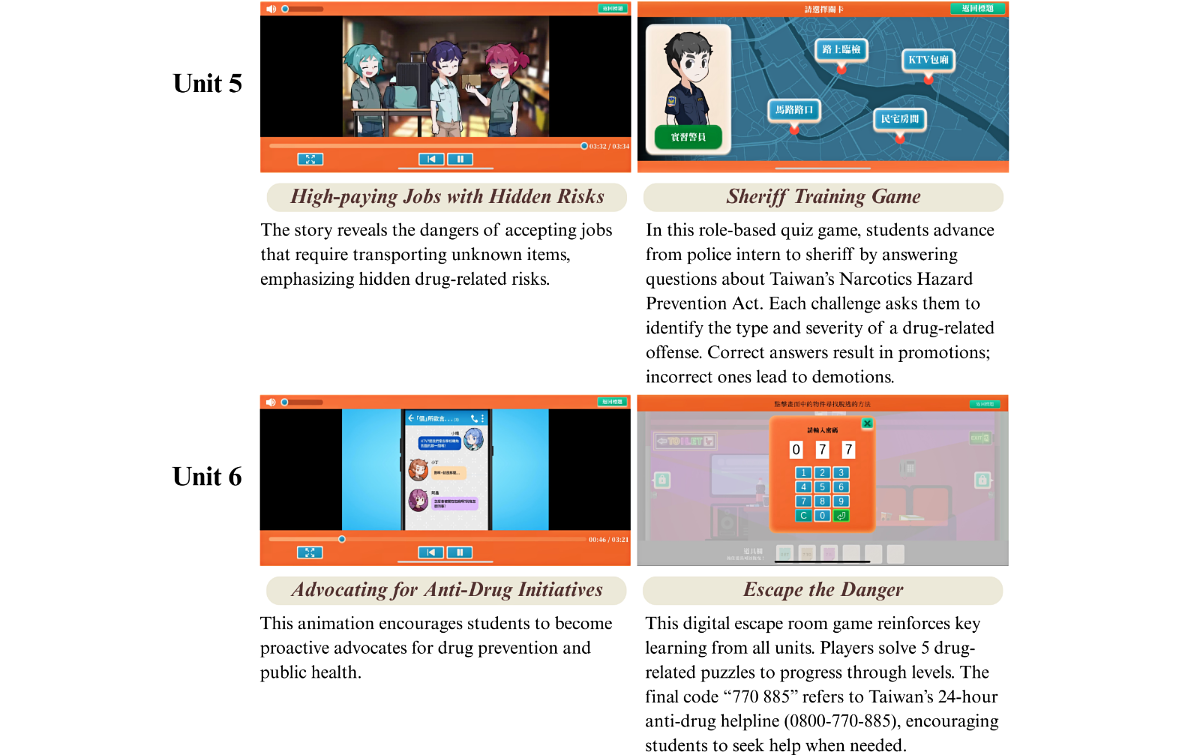
Screenshots of animations and corresponding web-based games from the interactive digital intervention (IDI) program (Units 5-6).

Each unit’s learning objectives, instructional material, and corresponding outcome variables are presented in Multimedia Appendix 1. Representative screenshots of the educational materials are presented in Figures 2-4. The program content aligns closely with established health literacy frameworks and life skills theories. To enhance accessibility, all digital materials were made available for download via Google Play (Google LLC) and the App Store (Apple Inc), thereby facilitating convenient access through smartphones or tablets for both students and educators.

Following the selection of the 5 experimental schools, the research team engaged with each school's administrative personnel and health education teachers to present a comprehensive overview of the study's objectives, methodology, and implementation procedures. Upon receiving formal approval to conduct the research, recruitment information was disseminated to students, inviting them to participate voluntarily. An orientation session was subsequently organized to ensure that the health education teachers had a thorough understanding of the study's aims and the collaborative responsibilities required for successful implementation. Thereafter, a half-day professional development workshop was conducted to introduce the components of the IDI, with particular emphasis on digital educational materials designed to enhance targeted outcome variables. Teachers were instructed to employ a combination of instructional approaches, including independent and classroom-based teaching, facilitated group discussions, and a culminating creative assignment, such as students producing short videos demonstrating effective refusal skills.

In the TD group, the students received a standard textbook-based drug education curriculum without interactive digital content. Generally, the didactic education in Taiwan is a conventional health education course that includes two 50-minute sessions on substance use prevention. The content of the standard textbook-based drug education curriculum included the harmful effects of using substances (tobacco, alcohol, betel nut, and illicit drugs), a positive attitude toward substance use prevention, and refusal skills, as shown in Multimedia Appendix 2.

### Measurement Instruments

All participants completed a structured questionnaire at baseline and the posttest period. The instrument comprised 6 domains: demographic characteristics, illegal drug-related knowledge, perceived harmfulness, health literacy, refusal skills, and learner engagement. It was adapted from previously validated instruments commonly used in adolescent health education research [21,24,39] and reviewed by experts for content relevance and cultural appropriateness in the Taiwanese school context.

Knowledge was assessed using an 11-item scale that evaluated students’ understanding of the risks and consequences associated with illegal drug use [40,41]. Each item was scored dichotomously, with 1 point awarded for each correct response and 0 points assigned for incorrect or “do not know” answers. The Cronbach α was 0.64 at baseline and 0.78 at posttest, indicating acceptable internal consistency.

Perceived harmfulness was measured with 4 items assessing students’ perceptions of the health risks related to tobacco, alcohol, betel nut, and illegal drugs [24,42]. Each item was scored on a 5-point Likert-type scale, ranging from 1 to 5. The Cronbach a was 0.70 at baseline and 0.73 posttest.

Health literacy was evaluated using a 17-question instrument encompassing 3 subdomains: functional, critical, and communicative literacy [17,43]. The internal consistency was robust, with a Cronbach α of 0.89 at baseline and 0.93 at posttest. Functional literacy comprises 5 questions, communicative literacy comprises 7 questions, and critical literacy encompasses 5 questions. This scale consists of 4-point Likert-type items, ranging from 1 (strongly disagree) to 4 (strongly agree). Items appear as 5 scenarios based on the logical flow of drug-use paragraphs, respectively.

Refusal skills were measured through 6 items assessing students’ confidence in resisting peer pressure, evaluating consequences, and assertively refusing inappropriate requests [24,44]. The Cronbach α was 0.89 at baseline and 0.94 at posttest.

Learner engagement was assessed using a 10-item scale covering both cognitive and emotional engagement [25,30]. This scale consisted of 5 cognitive and 5 emotional items, with Cronbach α values of 0.94 at baseline and 0.96 at the posttest.

In all variables, higher scores reflected more favorable outcomes.

### Statistical Analysis

Descriptive statistics were used to summarize demographic variables. *T* tests, chi-square, or Fisher exact tests were applied to examine group differences. Paired *t* tests were also conducted to evaluate intragroup changes from baseline to posttest. Given that schools were randomized at the cluster level, intraclass correlation coefficients were calculated for key outcome variables to assess within-cluster similarity. The intraclass correlation coefficient values for all outcome variables were below 0.12, suggesting that the influence of school-level clustering was likely minimal [45]. The generalized estimating equation (GEE) model was used to analyze time, group, and interaction effects on all outcome variables. All analyses were conducted using SPSS software (version 23.0; IBM Corp).

### Ethical Considerations

This study was reviewed and approved by the Research Ethics Committee of National Taiwan Normal University (202304HM008). Written informed consent was obtained from all participants and their legal guardians after the study purpose and procedures were explained. The research procedures followed the ethical standards of the responsible committee on human experimentation and the principles of the Declaration of Helsinki. All participant data were kept confidential and used solely for research purposes.

## Results

### Participant Characteristics

Table 1 presents demographic characteristics and background comparisons between participants in the IDI and TD groups. Significant differences were found between the 2 groups in terms of gender (χ²=5.7, *P*=.02) and age (t_766_=3.69, *P*<.001). However, the actual mean age difference between groups was less than 1 year, suggesting limited practical significance despite statistical significance due to the large sample size. No statistically significant differences were observed within groups between baseline and posttest with respect to parental education level, parenting style, and family structure.

**Table 1:** Background comparisons of the IDI^a^ and TD^b^ groups.

Variable		IDI group (n=305)	TD group (n=346)	Chi-square (*df*)/*t* test (*df*)	*P* value
**Gender^c^, n (%)**				5.7 (1)	.02^d^
	Male	150 (49.3)	201 (58.8)		
	Female	154 (50.7)	141 (41.2)		
Age^c^, mean (SD)		16.36 (2.80)	15.75 (0.74)	3.69 (649)	<.001
**Parental education^c^, n (%)**				1.0 (2)	.60
	Junior high school and below	47 (15.6)	62 (18.0)		
	Senior and vocational high school	116 (38.5)	136 (39.5)		
	College graduate or above	138 (45.8)	146 (42.4)		
**Parenting style, n (%)**				1.3 (1)	.28^d^
	Discuss together	251 (82.3)	296 (85.5)		
	Other	54 (17.7)	50 (14.5)		
**Family structure, n (%)**				2.6 (1)	.12^d^
	Live with parents	176 (57.7)	178 (51.4)		
	Other	129 (42.3%)	168 (48.6)		
**Prior personal substance use^c^, n (%)**				0.7 (1)	.40^d^
	No	198 (65.3)	237 (68.5)		
	Yes	105 (34.7)	109 (31.5)		
**Prior personal illegal drug use, n (%)**				N/A^e^	N/A
	No	305 (100.0)	346 (100.0)		
	Yes	0 (0.0)	0 (0.0)		
**Friends used to smoke, drink, and chew betel nut, n (%)**				0.3 (1)	.63^d^
	No	171 (56.1)	201 (58.1)		
	Yes	134 (43.9)	145 (41.9)		
**Friends have used illegal drugs^a^, n (%)**				0.2 (1)	.76^d^
	No	291 (98.6)	339 (98.3)		
	Yes	4 (1.4)	6 (1.7)		
**Family used to smoke, drink, and chew betel nut, n (%)**				0.4 (1)	.58^d^
	No	141 (46.2)	152 (43.9)		
	Yes	164 (53.8)	194 (56.1)		
**Family has used illegal drugs^c^, n (%)**				0.9 (1)	>.99^d^
	No	297 (100.0)	345 (99.7)		
	Yes	0 (0.0)	1 (0.2)		

^a^IDI: interactive digital intervention.

^b^TD: traditional didactic.

^c^Incomplete responses.

^d^Due to the expected count of cells being less than 5, Fisher's exact test was adopted.

^e^N/A: not available. Unable to calculate statistics.

Personal substance use was defined as the use of legal addictive substances such as tobacco, alcohol, and betel nut. Personal illegal drug use referred to the use of substances that are prohibited by law in Taiwan, including but not limited to marijuana, methamphetamine, and ketamine. Based on these definitions, the participants' histories of personal substance use did not significantly differ between the groups. Both groups had no reported prior illegal drug use.

Furthermore, no significant differences were observed between the IDI and TD groups regarding the substance use behaviors of friends and family members. Given the significant between-group differences in gender and age, these 2 variables were subsequently controlled for in the GEE analyses to ensure an accurate evaluation of the intervention effects.

### Intragroup Changes in Outcome Variables

Paired *t* tests comparing baseline and posttest scores within groups revealed that the IDI group had significantly greater improvements in the following variables: knowledge knowledge (t_304_=–5.23, *P*<.01), health literacy (t_304_=–3.18, *P*<.01), functional literacy (t_304_=–3.50, *P*<.01), critical literacy (t_304_=–2.79, *P*=.01), communicative literacy (t_304_=–2.26, *P*=.02), and learner engagement (t_304_=–3.40, *P*<.01), including cognitive engagement (t_304_=–2.20, *P*=.03) and emotional engagement (t_304_=–3.84, *P*<.01), except for perceived harmfulness and refusal skills. However, no significant intragroup differences were found in the TD group (Table 2).

**Table 2:** Paired sample *t* tests of the IDI^a^ and TD^b^ groups (within groups).

Variable				IDI group						TD group				
				Mean (SD)		*t* value (*df*)		*P* value		Mean (SD)		*t* value (*df*)		*P* value
**Knowledge**						–5.23 (304)		<.01				–0.36 (345)		.72
	Pretest			9.96 (1.23)						10.12 (1.13)				
	Posttest			10.39 (1.03)						10.15 (1.17)				
**Perceived harmfulness**						–0.96 (304)		.34				–0.40 (345)		.69
	Pretest			13.33 (2.00)						13.36 (2.06)				
	Posttest			13.47 (1.98)						13.42 (2.02)				
**Health literacy**						–3.18 (304)		<.01				0.06 (345)		.95
	Pretest			60.59 (6.11)						61.47 (5.91)				
	Posttest			62.05 (6.20)						61.45 (6.29)				
	**Functional literacy**					–3.50 (304)		<.01				–0.54 (345)		.59
		Pretest	17.60 (2.12)						18.01 (2.03)					
		Posttest	18.16 (2.14)						18.09 (2.10)					
	**Critical literacy**					–2.79 (304)		.01				0.75 (345)		.45
		Pretest	18.26 (1.99)						18.40 (1.85)					
		Posttest	18.66 (1.87)						18.30 (1.89)					
	**Communicative literacy**					–2.26 (304)		.02				0.09 (345)		.93
		Pretest	24.73 (2.93)						25.08 (2.94)					
		Posttest	25.22 (2.98)						25.06 (3.13)					
**Refusal skills**						–1.12 (304)		.26				0.48 (345)		.63
	Pretest			27.31 (3.98)						27.89 (3.66)				
	Posttest			27.63 (3.82)						27.76 (3.66)				
**Learner engagement**						–3.40 (304)		<.01				–0.48 (345)		.63
	Pretest			49.38 (12.20)						49.41 (12.93)				
	Posttest			52.78 (13.17)						49.88 (13.47)				
	**Cognitive engagement**					–2.20 (304)		.03				–0.32		.75
		Pretest	27.11 (6.44)						27.63 (7.04)					
		Posttest	28.24 (6.57)						27.79 (6.86)					
	**Emotional engagement**					–3.84 (304)		<.01				–0.57 (345)		.57
		Pretest	22.25 (7.31)						21.78 (7.54)					
		Posttest	24.54 (7.84)						22.11 (8.10)					

^a^IDI: interactive digital intervention.

^b^TD: traditional didactic.

### GEE Analysis

Table 3 presents the results of the GEE analyses, which examined the effects of group, time, and the group-by-time interaction on outcome variables after controlling for gender and age. Significant group-by-time interactions were observed for knowledge (coefficient=0.33, Wald χ^2^_1_=9.56; *P*<.01), overall health literacy (coefficient=1.49, Wald χ^2^_1_=8.46; *P*<.01), functional literacy (coefficient=0.48, Wald χ^2^_1_=6.96; *P*=.01), critical literacy (coefficient=0.52, Wald χ^2^_1_=9.21; *P*<.01), communicative literacy (coefficient=0.55, Wald χ^2^_1_=4.92; *P*=.03), overall learner engagement (coefficient=2.48, Wald χ^2^_1_=4.56; *P*=.03), and emotional engagement (coefficient=1.97, Wald χ^2^_1_=16.02; *P*<.01). In contrast, perceived harmfulness, refusal skills, and cognitive engagement did not show statistically significant group-by-time interactions.

**Table 3:** Results of the GEE^a^ analyses for outcome variables.

Variable				Coefficient (β)	Standard error		Wald χ^2^ (*df*)		*P* value
**Knowledge**									
	Group (IDI^b^ group)^c^			–0.15	0.09		3.12 (1)		.08
	Time (posttest)^d^			0.08	0.07		1.19 (1)		.28
	Group (IDI) × time (posttest)^e^			0.33	0.11		9.56 (1)		<.01
**Perceived harmfulness**									
	Group (IDI group)^c^			–0.07	0.16		0.22 (1)		.64
	Time (posttest) ^d^			0.04	0.12		0.11 (1)		.75
	Group (IDI) × time (posttest)^e^			0.16	0.18		0.83 (1)		.36
**Health literacy**									
	Group (IDI group)^c^			–1.12	0.47		5.66 (1)		.02
	Time (posttest)^d^			0.03	0.35		0.01 (1)		.94
	Group (IDI) × time (posttest)^e^			1.49	0.51		8.46 (1)		<.01
	**Functional literacy**								
		Group (IDI group)^c^	–0.44		0.16	7.63 (1)		.01	
		Time (posttest)^d^	0.09		0.12	0.57 (1)		.45	
		Group (IDI) × time (posttest)^e^	0.48		0.18	6.96 (1)		.01	
	**Critical literacy**								
		Group (IDI group)^c^	–0.23		0.14	2.55 (1)		.11	
		Time (posttest)^d^	–0.13		0.12	1.22 (1)		.27	
		Group (IDI) × time (posttest)^e^	0.52		0.17	9.21 (1)		<.01	
	**Communicative literacy**								
		Group (IDI group)^c^	–0.46		0.23	3.93 (1)		.05	
		Time (posttest)^d^	0.01		0.17	0.01 (1)		.94	
		Group (IDI) × time (posttest)^e^	0.55		0.25	4.92 (1)		.03	
**Refusal skills**									
	Group (IDI group)^c^			–0.67	0.29		5.42 (1)		.02
	Time (posttest)^d^			–0.09	0.23		0.17 (1)		.68
	Group (IDI) × time (posttest)^e^			0.53	0.33		2.55 (1)		.11
**Learner engagement**									
	Group (IDI group)^c^			–0.40	1.07		0.14 (1)		.71
	Time (posttest)^d^			0.41	0.77		0.28 (1)		.60
	Group (IDI) × time (posttest)^e^			2.48	1.16		4.56 (1)		.03
	**Cognitive engagement**								
		Group (IDI group)^c^	–0.91		0.55	2.74 (1)		.10	
		Time (posttest)^d^	0.07		0.41	0.03 (1)		.86	
		Group (IDI) × time (posttest)^e^	0.86		0.62	1.92 (1)		.17	
	**Emotional engagement**								
		Group (IDI group)^c^	0.13		0.65	0.04 (1)		.85	
		Time (posttest)^d^	0.00		0.33	0.00 (1)		1.00	
		Group (IDI) × time (posttest)^e^	1.97		0.49	16.02 (1)		<.01	

^a^GEE: generalized estimating equation.

^b^IDI: interactive digital intervention.

^c^Reference group (group): traditional didactic (TD) group.

^d^Reference group (time): pretest.

^e^Reference group (group × time): TD group × pretest.

Overall, the results of the GEE analyses were largely consistent with the paired *t* test findings, with the exception of cognitive engagement, which was significant in the paired *t* test but did not reach statistical significance in the GEE analysis.

## Discussion

### Principal Results

The findings indicated that the IDI had a significant positive impact on students’ substance use prevention outcomes. Specifically, the IDI group demonstrated statistically significant improvements in knowledge, health literacy—including functional, critical, and communicative literacy—as well as learner engagement, encompassing both cognitive and emotional components. The GEE analyses further confirmed significant group-by-time interaction effects for knowledge, health literacy (functional, critical, and communicative health literacy), and emotional engagement. These findings provide empirical support for the use of interactive digital learning in enhancing students’ knowledge acquisition, critical thinking, and emotional involvement in health-related decision-making.

The intervention significantly improved students’ knowledge of drug use prevention, as evidenced by both the paired *t* test and GEE analyses. This finding aligns with prior research demonstrating that interactive, gamified educational approaches can effectively enhance adolescents’ understanding of substance-related risks and misconceptions [23,33,46]. Through scenario-based games and animated modules, students were exposed to realistic situations involving drug use, enabling them to better understand the consequences of their choices and address existing misunderstandings. Compared to the traditional didactic approach, this digital format likely facilitated deeper engagement and improved retention of information. These results underscore the potential of an IDI as a scalable and effective strategy for increasing knowledge in school-based substance use prevention programs.

Despite being a core component of the intervention, perceived harmfulness did not show significant improvement after the intervention. This result may be attributed to 2 critical reasons. First, it is likely that the students had already developed a strong awareness of the harmful effects of substance use prior to the intervention. In Taiwan, antidrug education is systematically introduced at the elementary and junior high school levels, which may have contributed to relatively high baseline scores and limited the room for further improvement at the senior high school level. The potential ceiling effect may have masked the actual attainments in this domain. Second, the TD group received a textbook-based substance use prevention program, which also emphasized the health risks associated with drug use. Because both groups were exposed to content related to perceived harmfulness, the difference in impact between the interactive digital and traditional instruction may have been minimized. This could explain why no significant group-by-time interaction was observed. Furthermore, these findings suggest that perceived harmfulness may be less sensitive to short-term interventions in populations already exposed to foundational drug education. Future research could explore more differentiated content, such as by emphasizing long-term consequences, social stigma, or peer influence, to enhance perceptual shifts in high-risk groups or those with lower baseline awareness.

The IDI produced significant improvements in students’ functional health literacy. Functional literacy refers to the ability to comprehend and apply basic health information, such as understanding the consequences of substance use or recognizing warning signs related to drug exposure [17]. This improvement suggests that the interactive digital curriculum effectively conveyed foundational drug-related knowledge in a format that students could easily absorb and apply. These findings are consistent with previous studies that emphasize the importance of functional health literacy in shaping adolescents’ health behaviors and decision-making [21,43]. For example, Lin et al (2021) [21] found that enhancing functional health literacy significantly contributed to improved drug-use prevention behaviors among junior high school students. In this study, the interactive design—featuring animations, quizzes, and scenario-based games—may have facilitated greater engagement with content and reinforced learning through repetition and immediate feedback. Moreover, the accessibility of the program via mobile devices may have supported students' independent review of content, further solidifying their understanding. These results underscore the importance of delivering basic health education through digital media, particularly when targeting foundational knowledge essential for informed health decisions.

The intervention also led to a significant enhancement in students’ critical health literacy, which reflects their ability to analyze, evaluate, and apply health-related information to real-life situations. This competence is essential for navigating complex social environments, resisting peer pressure, and making autonomous decisions regarding substance use [17]. Game-based learning elements, such as scenario-based decision-making, interactive dialogues, and problem-solving tasks, may have been particularly effective in fostering these higher-order cognitive skills. For instance, one of the program modules featured a simulated conversation in which students had to evaluate a peer’s suggestion to use drugs, encouraging them to apply critical thinking in a realistic context. These findings are consistent with prior research suggesting that adolescents who possess strong critical health literacy are better equipped to reject misinformation and are less likely to engage in substance use [21,43]. Moreover, the engaging nature of digital content may promote deeper cognitive processing and sustained attention, both of which are conducive to developing critical literacy. Given the growing complexity of drug-related information, especially regarding emerging substances, enhancing students’ critical thinking and appraisal skills is increasingly crucial for equipping them to practice critical judgment in health-related contexts.

Significant improvements were also observed in students’ communicative health literacy, indicating their ability to interpret and effectively apply health information through interpersonal interactions, such as discussing substance use risks, negotiating peer pressure, and articulating personal boundaries [18,19]. The components of this intervention program, including simulated conversations and interactive group discussions, distinctly facilitate meaningful practice in communication skills. Through virtual dialogues and interactive scenarios, students can respond to situations involving peer suggestions related to substance use or pressures to engage in risky behaviors. Previous research suggests that scenario-based instruction effectively enhances communicative skills by enabling learners to engage in real-life situations, rehearse appropriate responses, and develop effective communication strategies [47]. A recent review underscored the effectiveness of interactive learning methods, including scenario simulations and role-playing delivered through digital platforms (eg, mobile apps and virtual reality), in enhancing adolescent health literacy. These approaches encourage learner engagement and facilitate better retention and application of health-related knowledge [48]. Consistent with these findings, the digital curriculum used in this study aligns with adolescents' preferences for interactive learning, effectively enhancing their health-related communication skills.

No significant improvement was observed in students’ refusal skills, which may be attributed to 2 possible reasons. First, students in both the IDI and TD groups had relatively high baseline scores, likely due to previous exposure to textbook-based drug prevention education through existing national health education curricula. In Taiwan, these programs typically cover well-established substances of concern, such as tobacco, alcohol, betel nut, ketamine, and methamphetamine. Refusal skills have long been a central component of drug prevention policies and are widely covered in elementary and junior high school programs through textbooks and school campaigns [49]. This long-term policy emphasis may have led to widespread existing skills, creating a ceiling effect that limits the possibility for further measurable progress. Second, while the content delivery methods varied, both the IDI and TD groups received instruction on refusal skills. In the TD group, teaching was presented in a conventional, lecture-based format over 2 class sessions, often complemented by brief role-play activities. This structure facilitates foundational knowledge transfer but restricts opportunities for students to revisit or deepen their skills. In contrast, the IDI group accessed refusal skill content repeatedly and in a more interactive format through animations and game-based scenarios. However, when students already possess a solid baseline understanding, the added benefit of digital interactivity may be less pronounced. Furthermore, refusal skills represent only one dimension of the larger life skills framework. The IDI curriculum was particularly effective in enhancing students’ health literacy, which includes critical thinking, evaluating information, and decision-making—skills that can have a wider and more lasting impact than just focusing on individual behavioral techniques. Although prior studies have highlighted the effectiveness of refusal skills training in reducing substance use [50-52], these findings suggest that the incremental benefit of interactive digital methods may be limited when foundational competencies are already in place. Future interventions could consider personalizing refusal skill activities based on students’ self-efficacy levels or incorporating more complex, socially embedded scenarios to challenge and refine their real-world application of these skills.

The intervention led to a notable increase in learner engagement, particularly in the emotional dimension. Engagement is crucial for educational outcomes, as it indicates students’ cognitive and emotional involvement in the learning process [25,30]. The interactive digital format utilized in this study—featuring engaging scenarios, challenge-based tasks, and immersive storytelling—likely contributed to increased student interest and motivation. Emotional engagement was particularly responsive to the intervention, as indicated by significant improvements in both statistical models. This can be attributed to the affective aspects of interactive digital learning, such as visual stimulation, narrative-driven challenges, and the rewarding experience of completing tasks. Previous research has supported that emotional engagement enhances learning persistence and memory retention, especially in digital environments [46,53]. Notably, while the paired *t* test indicated a significant increase in cognitive engagement, this was not reflected in the GEE model. This difference may result from the GEE model’s adjustment for covariates and group-by-time interaction effects, providing more conservative estimates than simpler tests. As GEE accounts for covariates and group-by-time interactions, we prioritized its findings in the interpretation. One possible explanation is that cognitive engagement, which involves sustained attention, deep processing, and strategic learning behaviors, may require longer or more individualized support to be fully activated. Studies have suggested that combining digital platforms with teacher guidance or peer collaboration can further promote cognitive involvement [30].

The findings of this study provide empirical support for the use of IDIs in preventing substance use among senior high school students. The intervention effectively enhanced knowledge, health literacy, and learner engagement—particularly emotional engagement. These outcomes demonstrate the value of integrating interactive and immersive learning strategies into substance prevention programs, especially in addressing the cognitive and affective dimensions of learning.

### Limitations

Several limitations should be noted. First, the relatively short duration of the IDI intervention and the absence of behavioral outcome measures may have limited our ability to evaluate long-term effectiveness. Second, high baseline scores in perceived harmfulness and refusal skills suggest a potential ceiling effect, which may have reduced the measurable impact of the program. Additionally, the TD group received textbook-based instruction covering critical prevention concepts, which may have diminished the effectiveness of the digital intervention when intergroup comparisons were conducted. Third, although the study utilized validated instruments with acceptable internal consistency, other psychometric properties, such as construct validity, test-retest reliability, and formal cultural adaptation, were not fully assessed. Fourth, teachers in the IDI group received only brief training to ensure proper operation of the digital materials, which may have introduced performance bias and variability in delivery. While this is a methodological limitation, it also reflects real-world school conditions. Importantly, this kind of training provides a foundation for teacher readiness, and standardized training should be considered essential for future policy implementation and large-scale adoption. These limitations should be considered when interpreting the study’s findings and in designing future research.

### Implications

Despite these limitations, the study provides valuable implications for practice. Integrating digital learning tools into school-based prevention programs can enhance student engagement and reinforce critical competencies related to drug refusal and health decision-making. However, outcomes such as refusal skills and perceived harmfulness showed no significant change, likely due to high baseline levels from prior exposure to national drug prevention curricula. This indicates that digital interventions might be more effective in enhancing foundational literacy and motivation than in altering well-established attitudes or behavioral skills. Future research could explore long-term effects through follow-up assessments, incorporate peer-led or teacher-facilitated components to strengthen communication outcomes, and tailor interventions based on students' baseline competencies to maximize their impact.

### Conclusions

This study demonstrated that an IDI can enhance certain drug use prevention outcomes, particularly students’ knowledge, health literacy, and learner engagement. However, some outcomes, such as refusal skills and perceived harmfulness, did not show significant change, possibly because of high initial levels or overlapping content in both groups. These findings highlight the potential of technology-enhanced intervention in promoting health-related learning. By integrating interactive storytelling, scenario-based tasks, and mobile accessibility, the intervention successfully engaged students both cognitively and emotionally, facilitating a deeper understanding and retention of drug prevention concepts. The findings support the incorporation of digital tools into school-based health education, offering a flexible and scalable approach to adolescent substance use prevention. Caution is advised when generalizing these results as evidence of overall effectiveness, and future research may build on these findings by extending intervention duration, incorporating peer interaction or teacher facilitation, and evaluating long-term behavioral outcomes further to enhance the impact of digital health literacy programs.

## Appendix

Multimedia Appendix 1 Learning objectives, materials, and outcome variables of the intervention program.

Multimedia Appendix 2 The content of standard textbook-based drug education curriculum.

## Abbreviations

GEE: generalized estimating equation
IDI: interactive digital intervention
TD: traditional didactic

## Data Availability

The data sets generated or analyzed in this study are available from the corresponding author upon reasonable request.
