# Supplementary material for: Effectiveness of an Interactive Digital Intervention Program on Knowledge, Health Literacy, and Learner Engagement in Senior High School Students: Intragroup and Intergroup Comparison of 2 Teaching Models
Source: J Med Internet Res. 2025 Sep 3;27:e76109. doi: 10.2196/76109 (PMC12444212; doi:10.2196/76109)
Supplement: Multimedia Appendix 1 [file jmir_v27i1e76109_app1.docx]

**Multimedia Appendix 1.** Learning objectives, materials, and outcome variables of the intervention program.

| Unit | Learning objectives | Educational Materials | Animation | Game | Outcome Variables |
| --- | --- | --- | --- | --- | --- |
| 1. | 1. Increase awareness of the health risks and seriousness linked to the use of addictive substances, and encourage a responsible attitude towards substance use. 2. Identify situations of prescription drug misuse, utilize decision-making skills, and implement avoidance strategies. | Materials aim to help participants understand the proper use of addictive substances and provide strategies for substance abuse prevention and management. These materials engage learners in cognitive understanding of the risks associated with drug use and in developing skills to resist misuse. | *The Deadly Temptation of Addiction* – This animation illustrates the consequences of substance misuse, highlighting the health risks and the decision-making process necessary to avoid addiction. | *Addiction Bingo* – This interactive game assists participants in recognizing addictive substances and their related symptoms, enhancing their understanding of addictive behaviors and preventive strategies. | - Knowledge acquisition regarding the health risks of addiction - Functional literacy in recognizing addiction symptoms - Critical literacy in applying decision-making strategies - Learner engagement in the interactive learning process - Development of refusal skills |
| 2. | 1. Acquire an in-depth understanding of common drug types, including the top five commonly used drugs^a^ and e-cigarettes. 2. Cultivate the ability to assess and recognize hazardous situations related to drug exposure. | The materials aim to inform participants about the different types of drugs and their associated risks while also providing tools to assess and navigate situations involving potential drug exposure. These materials promote critical thinking by assisting individuals in identifying risky situations and developing effective responses. | *Awareness and Decision*-*making in Drug Encounters* – This animation showcases various drug-related situations, assisting participants in recognizing and evaluating risky encounters involving drugs. | *Dangerous Situation Slot Machine* – In this game, participants evaluate various scenarios and must determine whether they indicate drug exposure, enhancing their ability to recognize and respond to such situations. | - Knowledge of various drug types, including new psychoactive substances - Critical literacy in evaluating dangerous situations - Communicative literacy in expressing concerns regarding drug exposure - Learner engagement through interactive scenario-based learning - Refusal skills development in drug-related contexts |
| 3. | 1. Understand the effects of drugs on physical and mental health. 2. Learn to identify distress signals and respond appropriately to drug exposure emergencies. 3. Understand the stages of addiction and its progression. | The materials emphasize providing a comprehensive understanding of the effects of drug use on both physical and mental health, along with guidance on recognizing and responding to drug-related distress signals. | *The Addiction Journey Scenario* – This animation depicts the progression of addiction, highlighting the health and psychological effects linked to prolonged drug use. | *Drug Connection Match* – This interactive game enables participants to match various drugs with their health effects, enhancing their understanding of addiction stages and the influence of drugs on physical and mental health. | - Knowledge of the physical and mental effects of drugs - Functional literacy in recognizing addiction stages - Critical literacy in identifying distress signals and emergency responses - Communicative literacy in managing drug-related emergencies - Learner engagement in understanding the addiction process |
| 4. | 1. Apply life skills to make informed decisions and adopt healthy behaviors for refusing drugs. 2. Use refusal skills to withstand peer pressure and drug-related invitations. | These materials are intended to assist participants in developing and applying life skills that empower them to make informed decisions regarding substance use and to decline drugs when offered by peers effectively. | *What Would You Think?* – This animation assists participants in navigating decision-making processes when drugs are offered, enhancing their confidence in resisting peer pressure. | *Myth Detective* – This game challenges participants to identify and dispel common myths about drugs, enabling them to practice refusal skills in hypothetical drug-related scenarios. | - Critical literacy in debunking drug-related myths - Communicative literacy in resisting peer pressure - Learner engagement through decision-making simulations - Development of decision-making and refusal skills |
| 5. | Foster critical thinking skills to challenge and rectify common misconceptions about drugs. | The materials aim to engage participants in critically evaluating and correcting misconceptions about drugs while providing a platform for developing informed, evidence-based decision-making. | *High-paying Jobs with Hidden Risks* – This animation prompts participants to critically assess seemingly safe or appealing situations that may harbor hidden drug-related risks, fostering awareness of potential dangers. | *Sheriff Training Game* – This game assesses participants' understanding of drug-related laws and common misconceptions, promoting critical thinking about illegal drug activities and their associated risks | - Knowledge acquisition regarding drug laws and risks - Functional literacy in recognizing and addressing drug myths - Critical literacy in evaluating potential risks in various situations - Learner engagement through interactive and scenario-based learning - Refusal skills in drug-related contexts |
| 6. | Develop advocacy skills to express an anti-drug stance and enhance public health awareness effectively. | These materials are designed to help participants develop and utilize advocacy skills, enabling them to effectively articulate their stance on drug prevention and support public health initiatives. | *Advocating for Anti-Drug Initiatives* – This animation enables participants to express anti-drug messages and become proactive advocates for drug prevention and public health. | *Escape the Danger* – In this game, participants utilize their advocacy skills to navigate challenging drug-related situations, emphasizing the significance of seeking help and making informed decisions. | - Functional literacy in advocating for drug prevention - Critical literacy in promoting public health and anti-drug awareness - Learner engagement in advocacy and health promotion activities - Refusal skills in advocating for individual and community health |

^a^ They were referenced from the study by Huang et al., (2023).
