# Supplementary material for: Effectiveness of an Interactive Digital Intervention Program on Knowledge, Health Literacy, and Learner Engagement in Senior High School Students: Intragroup and Intergroup Comparison of 2 Teaching Models
Source: J Med Internet Res. 2025 Sep 3;27:e76109. doi: 10.2196/76109 (PMC12444212; doi:10.2196/76109)
Supplement: Multimedia Appendix 2 [file jmir_v27i1e76109_app2.docx]

**Multimedia Appendix 2.** The content of standard textbook-based drug education curriculum.

| Unit | Learning objectives | Topics discussed | Program Delivery (Didactic) | Assessment of Content Knowledge and Outcomes |
| --- | --- | --- | --- | --- |
| 1. | 1. Increase awareness of the health risks and seriousness linked to the use of addictive prescription drugs. 2. Identify situations of prescription drug misuse, and implement avoidance strategies. 3. Encourage a responsible attitude and behaviors toward medication use. | - Definitions of substance abuse and addictive prescription drugs - Drug classifications (opioids, stimulants, sedatives) and health risks - Common sources of access and high-risk situations. - Strategies for avoiding addictive prescription drug use | - Teacher-led lectures - Textbook chapters - Group discussion - PowerPoint slides with case examples | - Learning sheet - Classroom Q & A |
| 2. | 1. Acquire an in-depth understanding of common drug types and other substance use. 2. Understand the effects of drugs on physical and mental health. 3. Cultivate the ability to assess and recognize hazardous situations related to drug exposure. 4. Apply life skills to make informed decisions and adopt healthy behaviors for refusing drugs. | - Physical, mental, and social consequences - Risk factors of substance use - Common substance use (e.g., e-cigarette, and drug types based on the Narcotics Hazard Prevention Act - Critical thinking skills to rectify common misconceptions about drugs. - Refusal skills to withstand peer pressure and drug-related invitations. | - Teacher-led lectures - Textbook chapters - Review of textbook case studies - PowerPoint slides with case examples - Group discussion | - Learning sheet - Unit test with scenario-based questions - Classroom Q & A |
